# Viral labeling of neurons synaptically connected to nucleus accumbens somatostatin interneurons

**DOI:** 10.1371/journal.pone.0213476

**Published:** 2019-03-07

**Authors:** Efrain A. Ribeiro, Alexander R. Nectow, Lisa E. Pomeranz, Mats I. Ekstrand, Ja Wook Koo, Eric J. Nestler

**Affiliations:** 1 Department of Neuroscience, Friedman Brain Institute, Icahn School of Medicine at Mount Sinai, New York, NY, United States of America; 2 Princeton Neuroscience Institute, Princeton, NJ, United States of America; 3 Laboratory of Molecular Genetics, The Rockefeller University, New York, NY, United States of America; 4 Department of Neural Development and Disease, Korea Brain Research Institute, Daegu, Republic of Korea; University of Texas at Austin, UNITED STATES

## Abstract

The nucleus accumbens, a key brain reward region, receives synaptic inputs from a range of forebrain and brainstem regions. Many of these projections have been established using electrophysiology or fluorescent tract tracing. However, more recently developed viral tracing techniques have allowed for fluorescent labeling of synaptic afferents in a cell type-specific manner. Since the NAc is comprised of multiple cell types, these methods have enabled the delineation of the cell type-specific connectivity of principal medium spiny neurons in the region. The synaptic connectivity of somatostatin interneurons, which account for <5% of the neurons in the region, has been inferred from electrophysiological and immunohistochemical data, but has not yet been visualized using modern viral tracing techniques. Here, we use the pseudorabies virus (PRV)-Introvert-GFP virus, an alphaherpes virus previously shown to label synaptic afferents in a cell type-specific manner, to label first order afferents to NAc somatostatin interneurons. While we find GFP(+) labeling in several well established projections to the NAc, we also observe that several known projections to NAc did not contain GFP(+) cells, suggesting they do not innervate somatostatin interneurons in the region. A subset of the GFP(+) afferents are c-FOS(+) following acute administration of cocaine, showing that NAc somatostatin interneurons are innervated by some cells that respond to rewarding stimuli. These results provide a foundation for future studies aimed toward elucidating the cell type-specific connectivity of the NAc, and identify specific circuits that warrant future functional characterization.

## Introduction

The nucleus accumbens, part of the ventral striatum, regulates motivation, reward, and aversion by integrating information from several distinct input pathways including glutamatergic inputs from the prefrontal cortex, ventral subiculum, amygdala, thalamus, and dopaminergic inputs from the ventral tegmental area (VTA) [1–3]. The NAc is primarily (~95%) comprised of principal GABAergic medium spiny neurons (MSNs) that express predominantly either D1 or D2 dopamine receptors, while the remaining neurons are GABAergic interneurons expressing somatostatin (SST)/nitric oxide synthetase-1 (NOS1), parvalbumin (PV), or the calcium-binding protein calretinin, and cholinergic interneurons expressing choline acetyltransferase. MSNs form the primary output pathways from the NAc that project to the ventral pallidum or midbrain.

Recent advances in genetic engineering have allowed researchers to modify neurotropic viruses to establish the cell type-specific connectivity of highly interconnected and heterogeneous brain regions such as the NAc [4,5]. The latter study used a modified rabies virus to trace inputs specifically to D1 or D2 expressing MSNs in the dorsal striatum using Cre-expressing transgenic mice. In this important paper, the group showed that, while D1 and D2 expressing neurons in the NAc receive the same set of inputs, certain inputs preferentially innervate one population of MSNs over the other. The use of distinct viral tracing systems combined with optogenetics has added a layer of complexity to the NAc circuitry, which has led to the formation of novel hypotheses regarding the function of specific neural projections to the NAc (e.g., [6–14]).

Among the GABAergic interneurons of the NAc, the SST/NOS1 expressing subtype have recently been shown to play an important role in regulating cocaine action [15,16]. Initial studies using electrophysiology and electron microscopy identified distinct chemical synapses (i.e., those containing dopamine or glutamate) on somatostatin expressing dendrites, suggesting that these cells likely receive the same range of inputs as MSNs in the NAc [17]. However, this question has not been addressed using contemporary viral tracing techniques.

Several groups in the field have used pseudorabies virus (PRV) to trace circuits in the brain via retrograde transport [18–20]. In a paper establishing the method, DeFalco and colleagues genetically modified the PRV-Bartha strain (termed Bartha2001) to depend upon expression of Cre recombinase for its replication [21]. The group then injected Bartha2001 into the hypothalamus of NPY-Cre mice to map the synaptic afferents to hypothalamic NPY(+) cells, cells that are involved in regulating food intake and satiety. They showed that Bartha2001 rapidly labeled afferents to Cre(+) cells in the region. In order to restrict off-target expression of the virus in Cre(-) cells, we introduced an “introvert” coding sequence requiring a double inversion event for successful coding of the genes required for viral replication (PRV-Introvert-GFP) [22]. As this system utilizes a single virus and labels synaptically connected neurons over the course of days, it offers an advantage over modified rabies virus systems for neuronal tracing.

In this study, we determined the time course of tracing with the PRV-Introvert-GFP virus and found that we could identify a progression of GFP(+) labeling in known NAc afferents over the course of 72 hr following injection into the NAc of SST-Cre mice. We identified a time point consistent with early synaptic spread, representing primary afferents to NAc somatostatin interneurons and found GFP expression in known NAc afferents. We quantified the amount of GFP staining in each of these regions and generated a weighted connectome for NAc somatostatin interneurons. By injecting the mice with cocaine prior to euthanasia, we identified activation of a subset of afferent neurons using c-Fos immunohistochemistry. Our data shed light on the complexity of the cell type-specific connectivity of the NAc, a highly interconnected brain region that is responsible for reward processing, and identify specific circuits that warrant further interrogation.

## Methods

### Animals

Male 8-12-week-old SST-Cre mice (on a C57BL/6J background) or wildtype C57BL/6J mice (all from Jackson Labs) were fed ad libitum and housed at 22–25°C on a 12 hr light/dark cycle. All experiments were performed in accordance with guidelines from the Society for Neuroscience. The institutional animal care and use committee at Mount Sinai specifically approved this study (laboratory animal protocol #08–0518; approval date 6/13/17). All mice were acclimated to vivarium conditions for at least one week prior to experimental manipulations. Cocaine-HCl (Sigma) was dissolved in sterile saline and administered IP at 20 mg/kg.

### Immunohistochemistry

Immunohistochemical experiments were performed on 100 μm thick brain sections at room temperature in 2 ml Eppendorf tubes on a rocking plate. We used sections of this thickness to more easily capture all labeled cells upon serially sectioning; imaging by confocal microscopy ensured that all labeled cells were nevertheless effectively captured and quantified. All experiments used the same blocking buffer which consists of 0.1% Triton in phosphate buffered saline (PBST) and 3% bovine serum albumin (BSA). Before staining, all sections were washed for 20 min with PBST and then blocked for 1 hr using the blocking buffer. Primary antibodies were added and the sections were kept at room temperature overnight. Sections were washed three times for 5 min with PBST. Secondary antibodies were added in blocking buffer and incubated for 1–3 hr. Sections were washed with PBST. DAPI was added to the final wash to stain nuclei. All sections were mounted using Prolong Gold Antifade (Life Technologies) and kept at 4°C for short-term storage and -20°C long-term storage.

### PRV-Introvert-GFP vector

We modified the alpha-Herpes virus Bartha strain’s UL23 locus to make the PRV-Introvert-GFP Cre-dependent vector used for trans-synaptic tracing. The Introvert genetic switch requires two Cre-mediated recombination events and subsequent splicing for the proper expression of viral thymidine kinase, which is essential for viral replication and for GFP expression. The conditional Introvert cassette eliminates low-level antisense transcription of an inverted open reading-frame, as has been observed in other putatively conditional tracing viruses. Thus, the conditional Introvert genetic switch prevents non-specific, and Cre-independent, tracing and represents a technical advance for connectivity mapping due to the increased control of cell type-specificity [22].

### Stereotaxic surgery

Mice were anesthetized with ketamine (100 mg/kg) and xylazine (10 mg/kg) in sterile saline injected IP. 33 gauge needles were used to bilaterally infuse alpha-Herpes virus into the NAc (AP = 1.5, ML = ±1.5, and DV = -4.4; 10° angle). An infusion volume of 0.5 μl was delivered using a 5 μl Hamilton syringe over the course of 5 min (at a rate of 0.1 μl/min). The infusion needle remained in place for at least 5 min after the infusions before removal to prevent backflow of the virus.

### Cell type-specific tracing with PRV-Introvert-GFP

By analyzing the spread of the virus in each mouse, we determined an optimal time point for first order tracing to synaptically connected neurons as previously described [17]. To establish a time point post-infection for rapid trans-synaptic tracing using the Introvert virus, we first performed an experiment using 8–12 week old SST-Cre mice. After infection, we collected brains from 2 mice at 5 time points including 12, 24, 36, 48, and 72 hr post-infection. At 72 hr, the mice showed no overt abnormalities, but all mice died by day 4–5, so longer time points were not included in the study. Mice were euthanized by deep chloral hydrate anesthesia and immediately perfused intracardially with PBS and 4% (wt/vol) PBS-buffered paraformaldehyde. Brains were dissected and post-fixed overnight at 4°C and then incubated in 30% sucrose for 48 hr. Brains were sectioned as above and free-floating sections were washed in PBST and then in blocking buffer for 1 hr. Chicken anti-GFP. Chicken anti-GFP (Aves; 1:1000) and/or mouse anti-c-Fos (Santa Cruz; SC-52) was added to the blocking solution and left overnight at room temperature. The next day, sections were washed in PBST then incubated in 1:750 of donkey anti-chicken Cy2 and/or donkey anti-mouse Cy3 (Immuno Research). All sections were counterstained and mounted with Prolong Gold solution including DAPI then subsequently imaged on a LSM 710/780 confocal microscope.

### Somatostatin subnetwork construction

Using the Allen Brain Atlas mouse connectome, we established a baseline set of inputs that we expected to detect if NAc somatostatin interneurons received monosynaptic inputs from every global afferent to the NAc [23]. While we expected up to 20% non-overlap due to the Allen Atlas’s methodology, we nevertheless filtered out any brain region that had a log10 power <3.5 as reported [23]. Starting with the global NAc connectome, we then determined which brain regions were present in the serial sections obtained. It was not possible to consistently section the posterior midbrain and hindbrain, so we excluded those regions from our global NAc network. We then collapsed regions according to confidence in our ability to determine anatomical sublocalizations of cells at high magnification. We included a collapsed measure for non-VTA midbrain/hindbrain to illustrate that somatostatin interneurons receive inputs from other nuclei in these posterior regions despite the fact that individual subregions could not be delineated.

## Results

### Synaptic tracing in SST-Cre mice using PRV-Introvert-GFP

**Fig 1:**
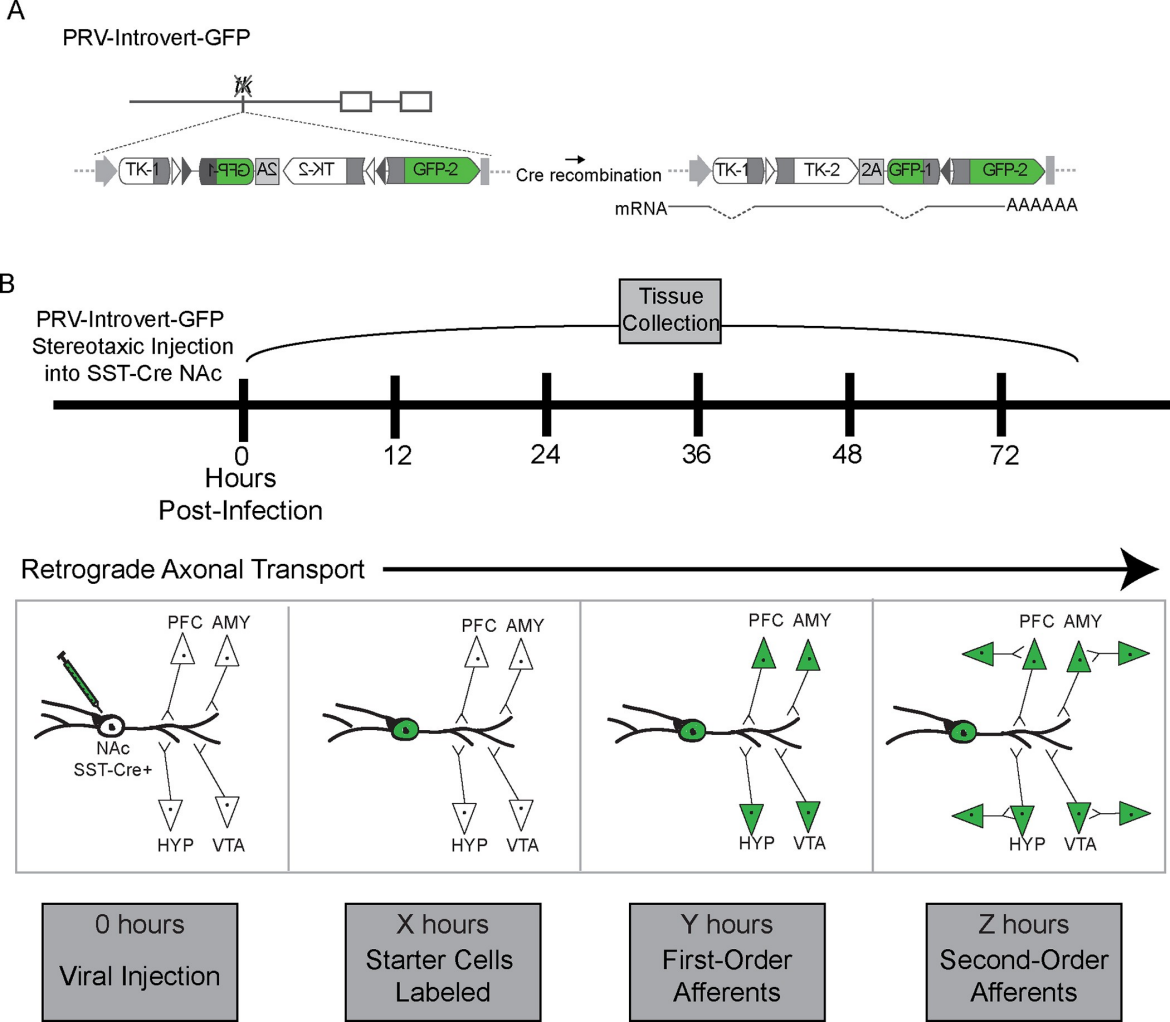
Experimental design for cell type-specific trans-synaptic tracing using PRV-Introvert-GFP, a modified conditional alpha-Herpes Bartha virus, in SST-Cre mice. **A)** Cre-dependent Introvert sequence used for cell type-specific tracing. **B)** Experimental timeline for determination of monosynaptic time point. We collected tissue from 2 mice every 12 hr and observed GFP expression in known NAc afferents. By collecting sections at serial time points, we determined time points at which viral labeling is first present in known NAc afferents. PFC–Prefrontal cortex, AMY–amygdala, HYP–hypothalamus, VTA–ventral tegmental area. These four regions shown as examples of a larger number of known input regions (see Fig 2).

We modified the alpha-Herpes virus Bartha strain’s UL23 locus to make the ‘Introvert’ Cre-dependent virus used for trans-synaptic tracing specifically in NAc SST-Cre cells. As previously established, we euthanized 2 mice every 12 hr after infection with PRV-Introvert-GFP for 72 hr and observed GFP expression in canonical NAc afferents in SST-Cre and wild-type C57BL/6J mice (Fig 1) [18,24]. Importantly, GFP expression was not observed at any time point in the wild-type C57BL/6J mice, showing that the Introvert coding sequence strongly limits Cre-independent viral propagation.

**Fig 2:**
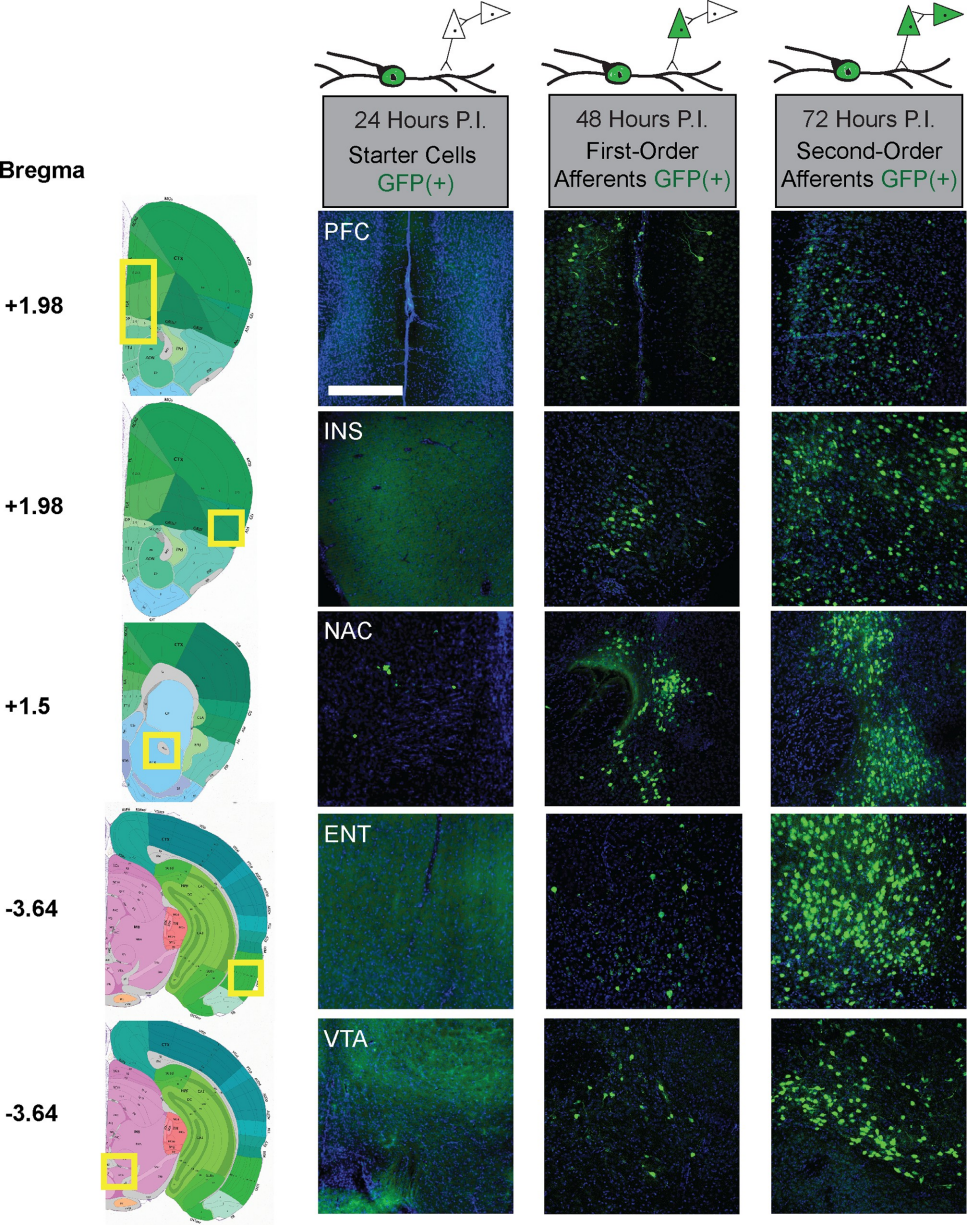
Time course for PRV-Introvert-GFP tracing. Representative sections from each 24 hr time point following infection with PRV-Introvert-GFP in SST-Cre mice. Scale bar = 250 μm. The first GFP(+) cells were identified in NAc at 24 hr post infection (P.I.). Representative sections shown for the following regions: PFC–prefrontal cortex, INS–insular cortex, PIR–piriform cortex, NAC–nucleus accumbens, ENT–entorhinal cortex, VTA–ventral tegmental area.

In SST-Cre mice, we found that at 24–36 hr after infection there were a small number of GFP(+) cells in the NAc, and no GFP(+) cells in any other regions. At 48 hr post infection, we were able to observe GFP(+) cells in multiple brain regions that constitute major projections to NAc (Fig 2). We did not observe further extension of this tracing until the 72 hr time point. Interestingly, in these mice, while GFP expression was prevalent throughout the brain, the tracing was clearly restricted to specific circuits as there were large brain areas devoid of detectable tracing. Further, there were regions of NAc that did not contain any GFP(+) staining at 72 hr, highlighting the segregation of convergent limbic pathways in the NAc, as recurrent labeling via feedback projections did not highlight the entire brain region.

### Global synaptic connectivity of NAc somatostatin interneurons

**Fig 3:**
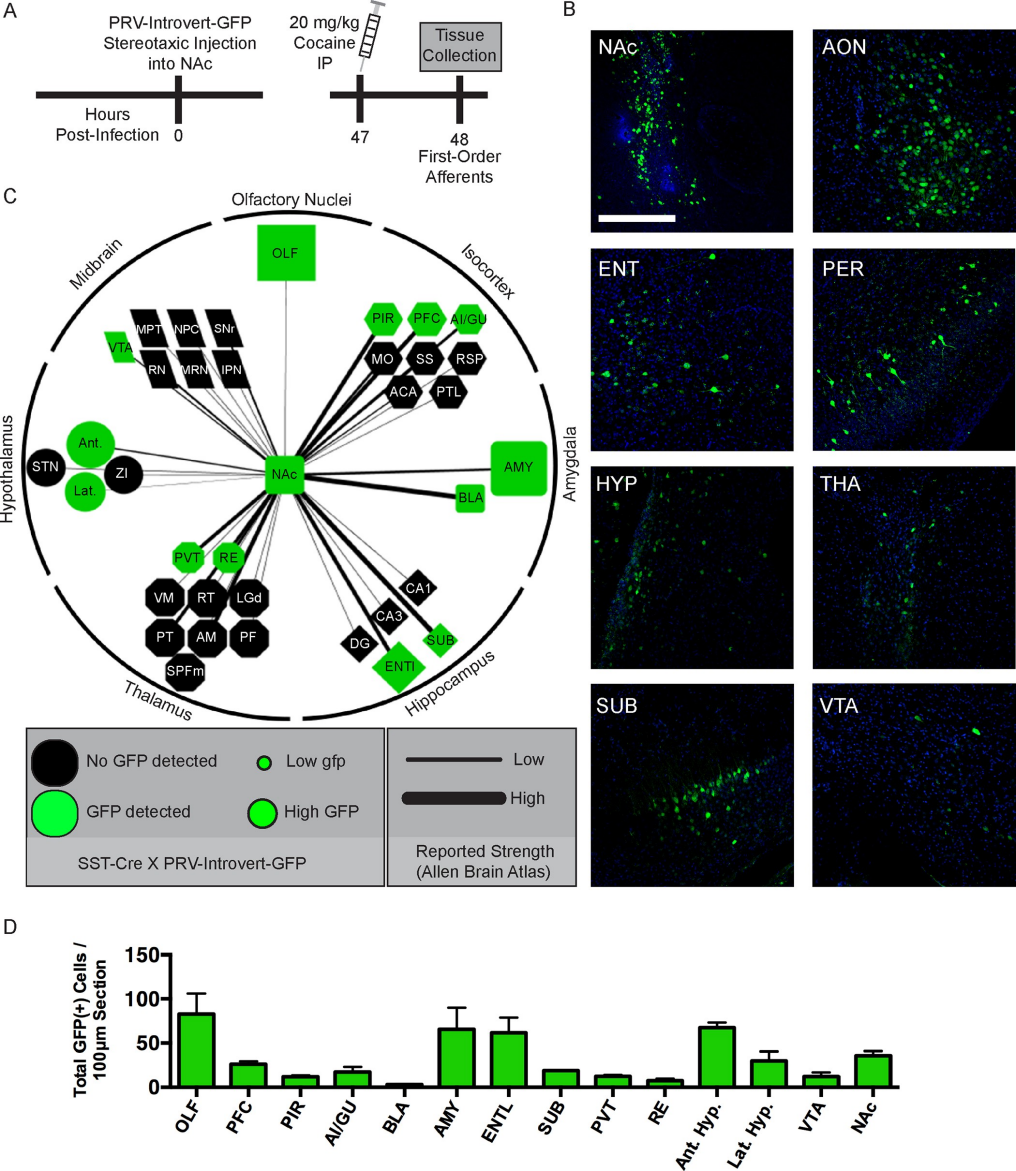
First order synaptic afferents to nucleus accumbens somatostatin interneurons. **A)** Experimental design for cell type-specific retrograde trans-synaptic tracing using PRV-Introvert-GFP in SST-Cre mice. The tracing data shown are from three mice, but we have replicated the tracing in three separate experiments with consistent findings obtained. **B)** Trans-synaptic tracing of afferents to NAc somatostatin interneurons. GFP is shown in Green. PFC–prefrontal cortex (IL+PL), AON/OLF–anterior olfactory nucleus, PIR–piriform cortex, AI/GU–anterior insular/gustatory cortex, BLA–basolateral amygdala, AMY–amygdala (non-BLA), ENTl–entorhinal cortex, HYP–hypothalamus, PVT–periventricular nucleus of the thalamus, RE–reuniens nucleus, SUB–ventral subiculum, VTA–ventral tegmental area, NAc–nucleus accumbens. TH–tyrosine hydroxylase (Red). Scale bar = 100 μm. **C)** Synaptic connectivity of NAc somatostatin interneurons. Baseline connectivity of mouse NAc was adapted from the Allen Brain Atlas (http://connectivity.brain-map.org/). Baseline connectivity strength for overall NAc was set at a maximum of 3.5 and used as a cutoff for counting a region as part of the global NAc connectome. The width of the line connecting each node/region to the NAc corresponds to the reported baseline connectivity strength for overall NAc. We observed GFP+ cells in green nodes shown in (C), while we did not detect GFP in regions shown in black nodes. The size of the green node represents the number of GFP+ cells that were labeled in each region after tracing in SST-Cre mice. For every region shown, the width of the line connecting the node to the NAc represents the reported/expected strength of each connection. (D) Quantification of results shown in network diagram.

Using this strategy, we designed an experiment to determine the synaptic connectivity of NAc somatostatin interneurons, and to quantify the activation of these afferents after acute exposure to cocaine (Fig 3A). We selected the 48 hr time point to analyze first-order synaptic tracing based on our initial time course experiment. This allowed us to visualize direct synaptic afferents to NAc somatostatin interneurons and quantify their relative strength based on the number of GFP(+) neurons labeled in each region. Since we had established the time point for primary synaptic tracing, we injected this cohort of SST-Cre mice with 20 mg/kg IP cocaine one hr prior to the timed euthanasia at 48 hr. This allowed us to determine what percentage of afferent neurons to NAc somatostatin interneurons are activated by acute exposure to cocaine using c-Fos immunohistochemistry, a marker of neuronal activation.

To visualize the results of the GFP(+) quantification, we assembled a network diagram using the reported strength of overall NAc connectivity [23] as well as our tracing results to represent the cell type-specific connectivity of NAc somatostatin interneurons (Fig 2B and 2C). As a blueprint for the diagram, we used the Allen Brain Atlas’s publication of the mesoscale mouse brain connectome to first denote major projections to NAc that we might expect to exhibit GFP(+) cells; this includes the prefrontal cortex, anterior olfactory nucleus, basolateral amygdala, thalamus, ventral subiculum, and VTA. All of the GFP+ regions identified in our experiments were among those captured by the Allen Brain Atlas. However, the converse is not true: several regions that project strongly to the NAc in the Allen Brain Atlas contained little or no GFP using the PRV-Introvert-GFP in SST-Cre mice. These regions included the CA1/CA3 subfields of the hippocampus, subthalamic nucleus, substantia nigra of the midbrain, and somatosensory and motor cortex. The lack of GFP(+) cells in these regions suggests that they synapse onto other cell types in NAc but not somatostatin interneurons.

### Activation of synaptic afferents to NAc somatostatin interneurons by cocaine

**Fig 4:**
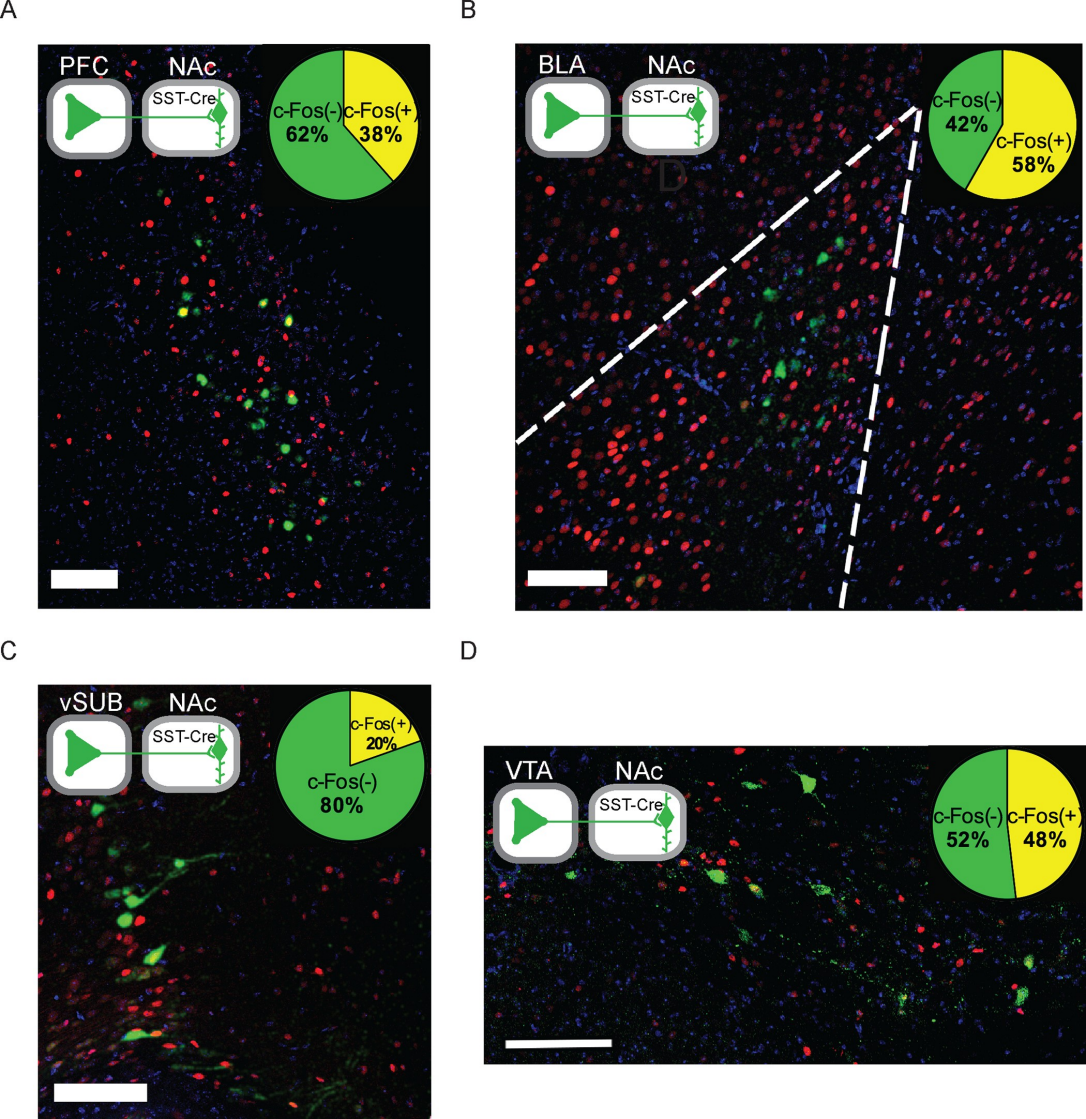
NAc somatostatin interneurons are innervated by first order afferents, a subset of which are activated during exposure to cocaine. Sections of prefrontal cortex (PFC), basolateral amygdala (BLA), ventral subiculum (vSUB), and VTA at the 48 hr monosynaptic time point are shown with c-Fos staining in red. Only a subset of GFP(+) cells in each region express c-Fos 60 min after exposure to cocaine. Scale bars = 100 μm.

We next sought to identify projection neurons to NAc somatostatin interneurons that are activated by acute cocaine exposure. We quantified c-Fos induction one hr after IP injection of 20 mg/kg cocaine in major afferents to NAc somatostatin interneurons, those originating in the prefrontal cortex, basolateral amygdala, ventral subiculum, and VTA (Fig 4). In each case, we found that a quarter to a half of GFP(+) neurons expressed c-Fos. These data show that at least some first-order afferents to NAc somatostatin interneurons are activated during exposure to cocaine.

**Fig 5:**
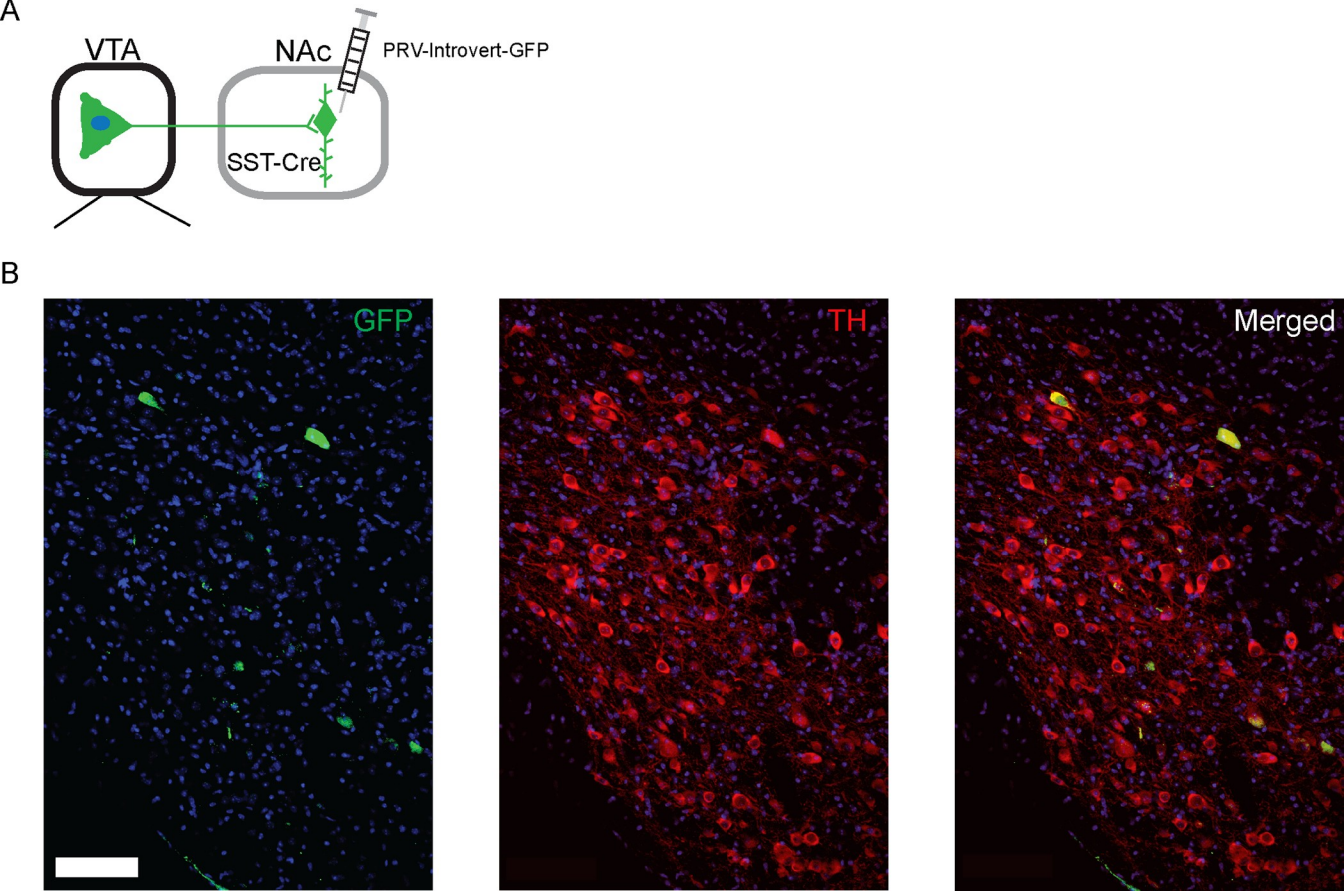
NAc somatostatin interneurons are innervated by VTA dopamine neurons. A section of VTA at the 48 hr monosynaptic time point is shown with TH staining in red. All GFP(+) cells identified express TH. Scale = 100 μm.

Since only a subset of VTA neurons that project to NAc somatostatin interneurons are c-Fos(+) one hr after exposure to cocaine, we wanted to address whether or not there are distinct cell types projecting to NAc somatostatin interneurons from within this region, as recent work has shown the involvement of a small GABAergic projection to NAc originating within the VTA [25]. We performed immunohistochemistry for tyrosine hydroxylase (TH) on GFP(+) cells in the VTA and found that all GFP(+) cells expressed TH, which categorizes the cells as dopaminergic (Fig 5). These data suggest that NAc somatostatin interneurons are not innervated by GABAergic cells originating in the VTA, only by TH(+) dopaminergic neurons.

## Discussion

In this study, we used PRV-Introvert-GFP, a modified alpha-Herpes Bartha virus containing an introvert coding sequence that improves cell type-specificity of transneuronal tracing. We injected the virus into the NAc of SST-Cre mice to identify synaptic afferents to somatostatin interneurons, a sparse cell type in this brain region. We found that retrograde tracing was successful in identifying synaptic afferents to these cells. While every region that projected to somatostatin interneurons was among the global afferents to the NAc as reported by the Allen Brain Atlas, several global afferents showed no detectable connections with somatostatin interneurons and, conversely, several connections to these interneurons were quantitatively stronger than that reported for the NAc overall. These results are consistent with the view that a cell type-specific connectome is necessary to fully elucidate the connectivity of the NAc and that some projections selectively innervate subsets of neuronal subtypes, including interneurons, in this brain region.

One caveat of these experiments is the possibility that there might be SST(+) populations of neurons elsewhere in the brain that project to the NAc. If this were the case, our labeling strategy would inadvertently label these afferents even if they are not synaptically connected to NAc SST interneurons. This is highly unlikely, since most SST expressing neurons are locally projecting GABAergic interneurons, and to our knowledge there is not a known projection of somatostatinergic cells to the NAc. This concern applies to other experimental systems dependent on Cre to drive tracing, where projections to a target region may contain Cre. Thus, further studies using this virus and other complementary tracing techniques are warranted to fully characterize the connectome of the NAc to rule out off-target labeling of projections that express the same driver as the starter cells being traced.

Our findings suggest several future experiments beyond neuronal tracing. In particular, our identification of specific circuits projecting to NAc somatostatin interneurons requires further electrophysiological studies to fully identify to what extent these inputs converge or diverge on neuronal subtypes within the NAc. Further, it will be important to use techniques such as immunohistochemistry or RNA-sequencing to identify the molecular phenotype of the projection neurons within each region, as it is possible that these projections may be heterogeneous with respect to their cell type and function. Nevertheless, direct innervation of NAc interneurons by numerous afferent brain regions suggests that these cells play a role not only in fine-tuning the intrinsic circuitry within the NAc, but also in controlling the ultimate impact of numerous afferent regions on activity of the NAc’s output MSNs. Moreover, as we show that several of the projections to NAc somatostatin interneurons are activated by acute cocaine exposure, it would be interesting to study the influence of these specific circuits per se on cocaine-related behaviors. For example, these findings raise the interesting possibility that cocaine’s net effect on the activity of NAc MSNs and hence on reward behavior occurs in part through activation of neurons throughout the limbic system—and not just effects intrinsic to the VTA→NAc pathway—and that this net effect is modulated by direct innervation of somatostatin interneurons by these afferent regions which then influence MSN activity.

As we were able to achieve trans-synaptic tracing in 48 hr, this method allows for the rapid identification and quantification of projections to specific cell types. Thus, it may be possible to detect differences in strength of connectivity following certain exposures in rodents, such as after prolonged drug administration or withdrawal. Other tracing methods require multiple rounds of surgeries and weeks of waiting time for fluorescent labeling. In our case, it may be possible to capture changes in circuitry more rapidly following these exposures. By performing these experiments at multiple timepoints, it should be possible to achieve better temporal resolution than traditional tracing methods. As just one example, with PRV-Introvert-GFP it would be possible to label and quantify afferents in a large population of mice across multiple time points in the context of an animal model of disease such as social defeat stress. With previous strategies, these experiments would be limited by resources, multiple surgeries and viruses, and time.

Our experiments shed light on the complexity of the cellular connectivity of the NAc by focusing on one subtype of GABAergic interneuron. In the future, it would be interesting to extend these studies still further to investigate the possible heterogeneity of inputs across a population of one cell type. As our system infects multiple starter somatostatin interneurons, we cannot appreciate differences within this population. Thus, it is possible that at the single-cell level the connectivity of somatostatin interneurons—or any other neuronal cell type—within a region may be different. New approaches aimed at elucidating these single-cell connectomes are better equipped to answer this important question that would have significant implications for our understanding of the brain’s circuitry and function.
